# Crystal structure of 4,5-bis­(3,4,5-tri­meth­oxy­phen­yl)-2*H*-1,2,3-triazole methanol monosolvate

**DOI:** 10.1107/S1600536814020911

**Published:** 2014-09-24

**Authors:** Nikhil Reddy Madadi, Narsimha Reddy Penthala, Shobanbabu Bommagani, Sean Parkin, Peter A. Crooks

**Affiliations:** a Department of Pharmaceutical Sciences, College of Pharmacy, University of Arkansas for Medical Sciences, Little Rock AR 72205, USA; b Department of Chemistry, University of Kentucky, Lexington KY 40506, USA

**Keywords:** crystal structure, hydrogen bonds, 1,2,3-triazole

## Abstract

The title compound, C_20_H_23_N_3_O_6_·CH_3_OH, was synthesized by [3 + 2] cyclo­addition of *(Z)*-2,3-bis­(3,4,5-tri­meth­oxy­phen­yl)acrylo­nitrile with sodium azide and ammonium chloride in DMF/water. The central nitro­gen of the triazole ring is protonated. The dihedral angles between the triazole ring and the 3,4,5-tri­meth­oxy­phenyl ring planes are 34.31 (4) and 45.03 (5)°, while that between the 3,4,5-tri­meth­oxy­phenyl rings is 51.87 (5)°. In the crystal, the mol­ecules, along with two methanol solvent mol­ecules are linked into an *R* ^4^_4_(10) centrosymmetric dimer by N—H⋯O and O—H⋯N hydrogen bonds.

## Related literature   

The synthetic procedure has been described by Madadi *et al.* (2014 ▶) and by Penthala *et al.* (2014 ▶). For structure-related activity, see: Young & Chaplin (2004 ▶); Pettit *et al.* (1995 ▶); Hsieh *et al.* (2005 ▶); Carr *et al.* (2010 ▶); Banimustafa *et al.* (2013 ▶); Demchuk *et al.* (2014 ▶).
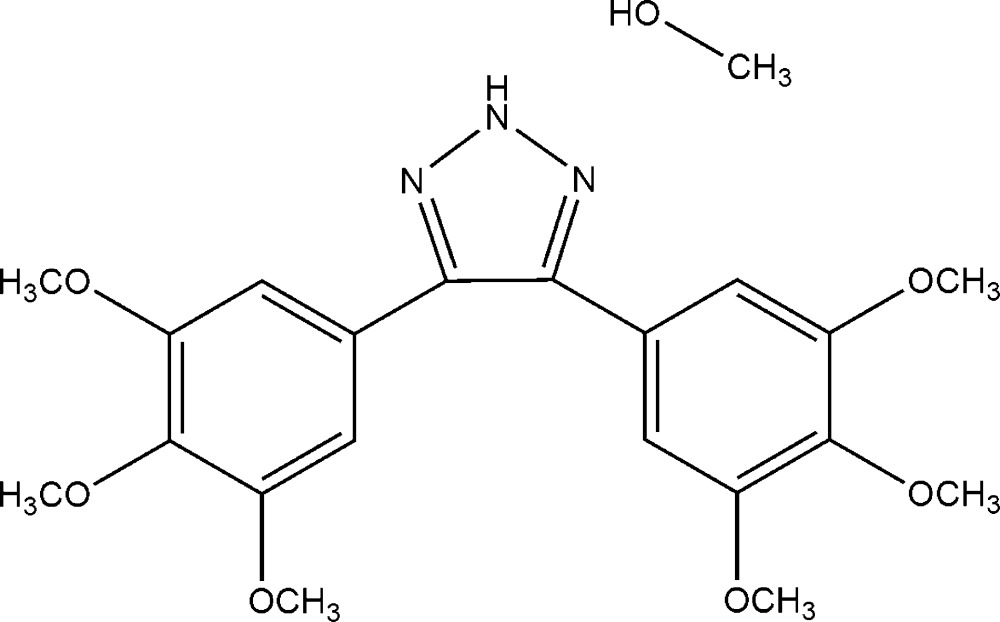


## Experimental   

### Crystal data  

C_20_H_23_N_3_O_6_·CH_4_O
*M* *_r_* = 433.45
Triclinic, 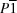

*a* = 10.1458 (1) Å
*b* = 10.6090 (1) Å
*c* = 11.0435 (2) Å
α = 89.5708 (6)°
β = 72.5903 (6)°
γ = 70.7146 (7)°
*V* = 1065.07 (2) Å^3^
*Z* = 2
Mo *K*α radiation
μ = 0.10 mm^−1^
*T* = 90 K
0.24 × 0.22 × 0.20 mm

### Data collection  

Nonius KappaCCD diffractometer
Absorption correction: multi-scan (*SADABS*; Sheldrick, 2008*a* ▶) *T*_min_ = 0.882, *T*_max_ = 0.970
28813 measured reflections
4887 independent reflections
3960 reflections with *I* > 2σ(*I*)
*R*_int_ = 0.037

### Refinement  

*R*[*F* ^2^ > 2σ(*F* ^2^)] = 0.045
*wR*(*F* ^2^) = 0.127
*S* = 1.05
4887 reflections
291 parameters
H atoms treated by a mixture of independent and constrained refinement
Δρ_max_ = 0.28 e Å^−3^
Δρ_min_ = −0.30 e Å^−3^

### 

Data collection: *COLLECT* (Nonius, 1998 ▶); cell refinement: *SCALEPACK* (Otwinowski & Minor, 2006 ▶); data reduction: *DENZO-SMN* (Otwinowski & Minor, 2006 ▶); program(s) used to solve structure: *SHELXS97* (Sheldrick, 2008*b* ▶); program(s) used to refine structure: *SHELXL2013* (Sheldrick, 2008*b* ▶); molecular graphics: *XP in *SHELXTL** (Sheldrick, 2008*b* ▶); software used to prepare material for publication: *SHELX* (Sheldrick, 2008*b* ▶).

## Supplementary Material

Crystal structure: contains datablock(s) global, I. DOI: 10.1107/S1600536814020911/sj5427sup1.cif

Structure factors: contains datablock(s) I. DOI: 10.1107/S1600536814020911/sj5427Isup2.hkl

Supporting information file. DOI: 10.1107/S1600536814020911/sj5427Isup3.cml

**Fig. 1.**
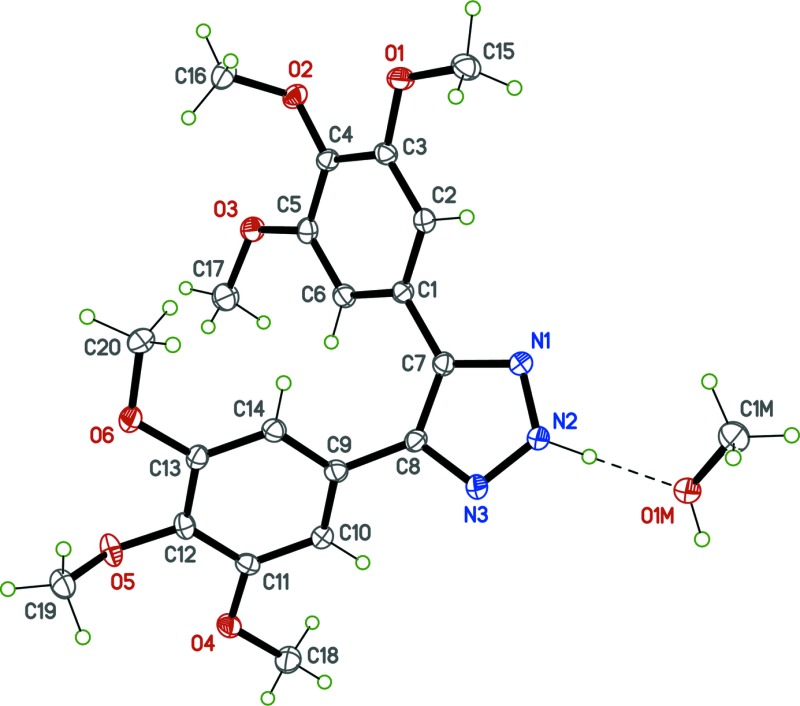
A view of the asymmetric unit of the structure with the atom numbering scheme. Displacement ellipsoids are drawn at the 50% probability level and a hydrogen bond is drawn as a dashed line.

. DOI: 10.1107/S1600536814020911/sj5427fig1.tif
A view of the asymmetric unit of the structure with the atom numbering scheme. Displacement ellipsoids are drawn at the 50% probability level and a hydrogen bond is drawn as a dashed line.

CCDC reference: 1025101

Additional supporting information:  crystallographic information; 3D view; checkCIF report

## Figures and Tables

**Table 1:** Hydrogen-bond geometry (Å, °)

*D*—H⋯*A*	*D*—H	H⋯*A*	*D*⋯*A*	*D*—H⋯*A*
N2—H2*N*⋯O1*M*	0.936 (17)	1.797 (18)	2.7303 (15)	174.2 (16)
O1*M*—H1*M*⋯N3^i^	0.84	1.97	2.8101 (15)	177

Symmetry code: (i) 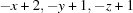.

## supplementary crystallographic information

### S1. Comment

Antimitotic agents are a major class of anticancer drugs that target the microtubules. There are three major binding sites on tubulin, namely the colchicine, taxane and vinca domains. Lately, anti-mitotic agents such as combretastatin A-4 that bind to the colchicine binding site are receiving much attention due to their potent anticancer and antiangiogenic properties. Combretastatin A-4 is a *cis* configured natural product extracted from the South African willow tree *combretum caffrum* (Pettit *et al.*, 1995). Its phosphate prodrug (CA-4P) is currently in phase 3 clinical trials for anaplastic thyroid cancer, and it has successfully arrested tumor growth in a wide spectrum of tumor models (Young and Chaplin, 2004). However, recent studies have reported the chemical instability of CA-4 due to *cis-trans* isomerization to the more thermodynamically stable, but less potent, *trans*-CA-4 isomer (Hsieh *et al.*, 2005). Recently, much research has been conducted to stabilize the *cis* configuration by replacing the ethylene bridge with heterocyclic ring systems, including thiazoles, tetrazoles, imidazoles, pyrazoles, oxazolones, triazoles, and furanones (Carr *et al.*, 2010; Banimustafa *et al.*, 2013; Demchuk *et al.*, 2014). One approach we are exploring is the replacement of the ethylene bridge in the *cis* stilbene scaffold with a triazole moiety to produce geometrically stable triazole analogs of CA4 with improved water solubility.

The title compound structure determination was performed to determine unequivocally the position of the hydrogen atom on the triazole ring system, which cannot be easily determined by NMR spectroscopy, and to obtain detailed information on the structural conformation of the molecule that may be useful in structure-activity relationship (SAR) analysis. The title compound was synthesized in two steps as described by Madadi *et al.*, 2014 and Penthala *et al.*, 2014).

In the first step, *(Z)*-2,3-bis(3,4,5-trimethoxyphenyl)acrylonitrile was synthesized by reacting 2-(3,4,5-trimethoxyphenyl)acetonitrile with 3,4,5-trimethoxybenzaldehyde in 5% NaOMe in methanol to afford the product in 85% yield. In the second step 4,5-bis(3,4,5-trimethoxyphenyl)-2*H*-1,2,3- triazole was synthesized by refluxing a mixture of *(Z)*-2,3-bis(3,4,5-trimethoxyphenyl)acrylonitrile, sodium azide and ammonium chloride in a DMF/water mixture to afford the desired product in 64% yield. The crystal structure of the compound indicates the presence of a 2*H* nitrogen on the triazole ring, *i.e.* protonation of the middle N atom. The molecules, along with two methanol solvent molecules, are linked into an *R*^4^_4_(10) centrosymmetric dimer by N—H···O and O—H···N intermolecular hydrogen bonds. The dihedral angles between the triazole ring and the two 3,4,5-trimethoxyphenyl ring planes are 34.31 (4)° and 45.03 (5)°, while that between the two 3,4,5-trimethoxyphenyl rings is 51.87 (5)°.

### S2. Experimental

A mixture of (*Z*)-2,3-bis(3,4,5-trimethoxyphenyl)acrylonitrile, sodium azide and ammonium chloride in a mole ratio of 1:3:3, respectively, was refluxed in 10% aqueous DMF for 5 hrs. The reaction was monitored by TLC. When the (*Z*)-2,3-bis(3,4,5-trimethoxyphenyl)-acrylonitrile strating material had completely disappeared, cold water was added and the mixture was stirred over 10–15 min, during which the final product precipitated out. The product was purified by flash column chromatography utilizing ethyl acetate/methanol to afford 4,5-bis(3,4,5-trimethoxyphenyl)-2*H*-1,2,3-triazole in 64% yield. Crystallization from methanol afforded a white crystalline product: 4,5-bis(3,4,5-trimethoxyphenyl)-2*H*-1,2,3-triazole methanolate, which was suitable for X-ray crystallographic analysis.

^1^H NMR (400 MHz, CDCl_3_-d): δ 3.93 (*s*, 3H, –OCH3), 6.87 (*d*, J = 8 Hz, 1H, ArH), 7.05 (*d*, J = 12 Hz, 2H, ArH),), 7.42 (*s*, 2H, ArH), 7.78 (*s*, 1H, ArH) *p.p.m.*^13^C NMR (400 MHz,CDCl_3_-d): δ 55.87, 55.96, 111.05, 127.31, 129.80, 129.89, 130.54, 132.60, 132.86, 149.15, 149.54 *p.p.m*. HRMS (ESI): *m/z* calcd for C_20_H_23_N_3_O_6_ [M—H] 350.0463; found 350.0480.

### S3. Refinement

H atoms were found in difference Fourier maps. Those bonded to carbon and oxygen were subsequently placed at idealized positions with constrained distances of 0.98 Å (RCH_3_), 0.95 Å (C_sp2_H) and 0.84 Å (OH), while the nitrogen-bound H atom position was refined. *U*_iso_(H) values were set to either 1.2*U*_eq_ or 1.5*U*_eq_ (RCH_3_, OH) of the attached atom.

### Crystal data

**Table:**

C_20_H_23_N_3_O_6_·CH_4_O	*Z* = 2
*M**_r_* = 433.45	*F*(000) = 460
Triclinic, *P*1	*D*_x_ = 1.352 Mg m^−^^3^
*a* = 10.1458 (1) Å	Mo *K*α radiation, λ = 0.71073 Å
*b* = 10.6090 (1) Å	Cell parameters from 11695 reflections
*c* = 11.0435 (2) Å	θ = 1.0–27.5°
α = 89.5708 (6)°	µ = 0.10 mm^−^^1^
β = 72.5903 (6)°	*T* = 90 K
γ = 70.7146 (7)°	Block, colourless
*V* = 1065.07 (2) Å^3^	0.24 × 0.22 × 0.20 mm

### Data collection

**Table:**

Nonius KappaCCD diffractometer	4887 independent reflections
Radiation source: fine-focus sealed-tube	3960 reflections with *I* > 2σ(*I*)
Detector resolution: 9.1 pixels mm^-1^	*R*_int_ = 0.037
φ and ω scans at fixed χ = 55°	θ_max_ = 27.5°, θ_min_ = 1.9°
Absorption correction: multi-scan (*SADABS*; Sheldrick, 2008*a*)	*h* = −13→13
*T*_min_ = 0.882, *T*_max_ = 0.970	*k* = −13→13
28813 measured reflections	*l* = −14→14

### Refinement

**Table:**

Refinement on *F*^2^	Primary atom site location: structure-invariant direct methods
Least-squares matrix: full	Secondary atom site location: difference Fourier map
*R*[*F*^2^ > 2σ(*F*^2^)] = 0.045	Hydrogen site location: difference Fourier map
*wR*(*F*^2^) = 0.127	H atoms treated by a mixture of independent and constrained refinement
*S* = 1.05	*w* = 1/[σ^2^(*F*_o_^2^) + (0.0731*P*)^2^ + 0.3615*P*] where *P* = (*F*_o_^2^ + 2*F*_c_^2^)/3
4887 reflections	(Δ/σ)_max_ < 0.001
291 parameters	Δρ_max_ = 0.28 e Å^−^^3^
0 restraints	Δρ_min_ = −0.30 e Å^−^^3^

### Special details

**Table:**

Experimental. The crystal was mounted with polyisobutene oil on the tip of a fine glass fibre, fastened in a copper mounting pin with electrical solder. It was placed directly into the cold stream of a liquid nitrogen based cryostat.Diffraction data were collected with the crystal at 90 K, which is standard practice in this laboratory for the majority of flash-cooled crystals.
Geometry. All e.s.d.'s (except the e.s.d. in the dihedral angle between two l.s. planes) are estimated using the full covariance matrix. The cell e.s.d.'s are taken into account individually in the estimation of e.s.d.'s in distances, angles and torsion angles; correlations between e.s.d.'s in cell parameters are only used when they are defined by crystal symmetry. An approximate (isotropic) treatment of cell e.s.d.'s is used for estimating e.s.d.'s involving l.s. planes.

### Fractional atomic coordinates and isotropic or equivalent isotropic displacement parameters (Å^2^)

**Table:**

	*x*	*y*	*z*	*U*_iso_*/*U*_eq_	
N1	0.60782 (12)	0.74167 (11)	0.56615 (11)	0.0185 (2)	
N2	0.73493 (13)	0.64285 (12)	0.54691 (11)	0.0195 (3)	
H2N	0.8264 (19)	0.6486 (17)	0.5010 (16)	0.023*	
N3	0.72437 (12)	0.53382 (11)	0.60414 (11)	0.0182 (2)	
O1	0.10274 (10)	1.12766 (9)	0.78293 (10)	0.0199 (2)	
O2	−0.09566 (10)	1.00928 (10)	0.79884 (9)	0.0203 (2)	
O3	−0.01750 (10)	0.74272 (10)	0.76182 (10)	0.0212 (2)	
O4	0.55195 (11)	0.11586 (9)	0.76505 (9)	0.0200 (2)	
O5	0.35131 (11)	0.22077 (10)	0.98936 (9)	0.0226 (2)	
O6	0.26188 (10)	0.48551 (10)	1.06686 (9)	0.0203 (2)	
C1	0.34889 (14)	0.77606 (13)	0.68391 (12)	0.0156 (3)	
C2	0.30637 (14)	0.91466 (13)	0.71055 (12)	0.0161 (3)	
H2	0.3785	0.9560	0.7006	0.019*	
C3	0.15713 (14)	0.99221 (13)	0.75193 (12)	0.0160 (3)	
C4	0.05096 (14)	0.93156 (13)	0.76591 (12)	0.0162 (3)	
C5	0.09440 (14)	0.79251 (14)	0.74196 (12)	0.0171 (3)	
C6	0.24333 (14)	0.71422 (14)	0.69882 (13)	0.0177 (3)	
H6	0.2728	0.6199	0.6798	0.021*	
C7	0.50685 (14)	0.69379 (13)	0.64045 (12)	0.0162 (3)	
C8	0.57985 (14)	0.56334 (13)	0.66514 (12)	0.0159 (3)	
C9	0.52294 (14)	0.47110 (13)	0.74808 (13)	0.0162 (3)	
C10	0.56894 (14)	0.33457 (13)	0.70935 (13)	0.0167 (3)	
H10	0.6368	0.2988	0.6270	0.020*	
C11	0.51433 (14)	0.25086 (13)	0.79276 (13)	0.0161 (3)	
C12	0.41268 (14)	0.30331 (14)	0.91361 (13)	0.0172 (3)	
C13	0.36502 (14)	0.44122 (14)	0.94987 (12)	0.0165 (3)	
C14	0.42169 (14)	0.52434 (14)	0.86842 (13)	0.0170 (3)	
H14	0.3916	0.6173	0.8946	0.020*	
C15	0.20801 (16)	1.19411 (14)	0.76486 (15)	0.0231 (3)	
H15A	0.2708	1.1587	0.8184	0.035*	
H15B	0.1565	1.2907	0.7887	0.035*	
H15C	0.2688	1.1785	0.6751	0.035*	
C16	−0.17239 (16)	1.02141 (16)	0.93261 (14)	0.0265 (3)	
H16A	−0.1634	0.9319	0.9605	0.040*	
H16B	−0.2765	1.0745	0.9489	0.040*	
H16C	−0.1297	1.0661	0.9799	0.040*	
C17	0.02045 (17)	0.60063 (15)	0.75393 (16)	0.0279 (3)	
H17A	0.0798	0.5621	0.6663	0.042*	
H17B	−0.0694	0.5778	0.7781	0.042*	
H17C	0.0771	0.5642	0.8119	0.042*	
C18	0.64600 (16)	0.06014 (15)	0.63927 (14)	0.0242 (3)	
H18A	0.6015	0.1072	0.5768	0.036*	
H18B	0.6593	−0.0354	0.6287	0.036*	
H18C	0.7417	0.0703	0.6259	0.036*	
C19	0.38996 (17)	0.19362 (16)	1.10416 (14)	0.0247 (3)	
H19A	0.4925	0.1338	1.0826	0.037*	
H19B	0.3256	0.1506	1.1593	0.037*	
H19C	0.3781	0.2780	1.1489	0.037*	
C20	0.19984 (16)	0.62778 (14)	1.10034 (13)	0.0220 (3)	
H20A	0.2772	0.6621	1.1044	0.033*	
H20B	0.1235	0.6476	1.1837	0.033*	
H20C	0.1564	0.6709	1.0358	0.033*	
O1M	1.00187 (11)	0.66705 (10)	0.42773 (10)	0.0244 (2)	
H1M	1.0849	0.6076	0.4149	0.037*	
C1M	1.01117 (17)	0.79571 (15)	0.45053 (15)	0.0269 (3)	
H1M1	0.9119	0.8626	0.4814	0.040*	
H1M2	1.0672	0.8206	0.3710	0.040*	
H1M3	1.0608	0.7922	0.5148	0.040*	

### Atomic displacement parameters (Å^2^)

**Table:**

	*U*^11^	*U*^22^	*U*^33^	*U*^12^	*U*^13^	*U*^23^
N1	0.0160 (5)	0.0170 (6)	0.0195 (6)	−0.0042 (4)	−0.0029 (4)	0.0023 (5)
N2	0.0155 (5)	0.0163 (6)	0.0228 (6)	−0.0052 (4)	−0.0011 (5)	0.0035 (5)
N3	0.0172 (5)	0.0164 (6)	0.0194 (6)	−0.0070 (4)	−0.0022 (4)	0.0033 (4)
O1	0.0167 (5)	0.0148 (5)	0.0270 (5)	−0.0046 (4)	−0.0063 (4)	0.0008 (4)
O2	0.0127 (4)	0.0229 (5)	0.0217 (5)	−0.0030 (4)	−0.0039 (4)	0.0033 (4)
O3	0.0166 (5)	0.0193 (5)	0.0281 (5)	−0.0088 (4)	−0.0047 (4)	0.0020 (4)
O4	0.0235 (5)	0.0141 (5)	0.0203 (5)	−0.0063 (4)	−0.0039 (4)	0.0011 (4)
O5	0.0322 (6)	0.0247 (5)	0.0171 (5)	−0.0179 (5)	−0.0077 (4)	0.0070 (4)
O6	0.0205 (5)	0.0186 (5)	0.0170 (5)	−0.0062 (4)	0.0003 (4)	0.0006 (4)
C1	0.0150 (6)	0.0161 (6)	0.0139 (6)	−0.0034 (5)	−0.0041 (5)	0.0031 (5)
C2	0.0161 (6)	0.0175 (7)	0.0160 (6)	−0.0074 (5)	−0.0052 (5)	0.0033 (5)
C3	0.0176 (6)	0.0147 (6)	0.0152 (6)	−0.0041 (5)	−0.0062 (5)	0.0030 (5)
C4	0.0138 (6)	0.0180 (7)	0.0156 (6)	−0.0034 (5)	−0.0053 (5)	0.0033 (5)
C5	0.0161 (6)	0.0219 (7)	0.0156 (6)	−0.0087 (5)	−0.0060 (5)	0.0042 (5)
C6	0.0188 (6)	0.0167 (7)	0.0178 (6)	−0.0069 (5)	−0.0054 (5)	0.0025 (5)
C7	0.0169 (6)	0.0164 (7)	0.0144 (6)	−0.0061 (5)	−0.0032 (5)	0.0014 (5)
C8	0.0142 (6)	0.0158 (6)	0.0154 (6)	−0.0043 (5)	−0.0022 (5)	−0.0007 (5)
C9	0.0147 (6)	0.0164 (7)	0.0179 (6)	−0.0050 (5)	−0.0061 (5)	0.0031 (5)
C10	0.0151 (6)	0.0172 (7)	0.0162 (6)	−0.0050 (5)	−0.0033 (5)	0.0012 (5)
C11	0.0167 (6)	0.0138 (6)	0.0195 (6)	−0.0055 (5)	−0.0080 (5)	0.0026 (5)
C12	0.0187 (6)	0.0196 (7)	0.0169 (6)	−0.0106 (5)	−0.0064 (5)	0.0050 (5)
C13	0.0140 (6)	0.0201 (7)	0.0151 (6)	−0.0062 (5)	−0.0040 (5)	0.0022 (5)
C14	0.0168 (6)	0.0153 (6)	0.0186 (6)	−0.0055 (5)	−0.0052 (5)	0.0015 (5)
C15	0.0216 (7)	0.0172 (7)	0.0318 (8)	−0.0079 (6)	−0.0089 (6)	0.0026 (6)
C16	0.0197 (7)	0.0293 (8)	0.0235 (7)	−0.0057 (6)	−0.0001 (6)	0.0031 (6)
C17	0.0246 (7)	0.0220 (8)	0.0373 (9)	−0.0128 (6)	−0.0047 (6)	0.0022 (6)
C18	0.0259 (7)	0.0177 (7)	0.0246 (7)	−0.0079 (6)	−0.0011 (6)	−0.0030 (6)
C19	0.0263 (7)	0.0267 (8)	0.0212 (7)	−0.0093 (6)	−0.0075 (6)	0.0089 (6)
C20	0.0215 (7)	0.0190 (7)	0.0191 (7)	−0.0030 (6)	−0.0017 (5)	−0.0013 (5)
O1M	0.0200 (5)	0.0177 (5)	0.0302 (6)	−0.0046 (4)	−0.0028 (4)	0.0040 (4)
C1M	0.0278 (7)	0.0227 (8)	0.0301 (8)	−0.0100 (6)	−0.0073 (6)	0.0020 (6)

### Geometric parameters (Å, º)

**Table:**

N1—N2	1.3243 (16)	C10—C11	1.3970 (18)
N1—C7	1.3473 (16)	C10—H10	0.9500
N2—N3	1.3339 (16)	C11—C12	1.4010 (19)
N2—H2N	0.936 (17)	C12—C13	1.4002 (19)
N3—C8	1.3455 (16)	C13—C14	1.3895 (18)
O1—C3	1.3628 (16)	C14—H14	0.9500
O1—C15	1.4301 (16)	C15—H15A	0.9800
O2—C4	1.3768 (15)	C15—H15B	0.9800
O2—C16	1.4330 (17)	C15—H15C	0.9800
O3—C5	1.3645 (16)	C16—H16A	0.9800
O3—C17	1.4236 (17)	C16—H16B	0.9800
O4—C11	1.3661 (16)	C16—H16C	0.9800
O4—C18	1.4268 (17)	C17—H17A	0.9800
O5—C12	1.3755 (16)	C17—H17B	0.9800
O5—C19	1.4358 (17)	C17—H17C	0.9800
O6—C13	1.3639 (16)	C18—H18A	0.9800
O6—C20	1.4326 (16)	C18—H18B	0.9800
C1—C2	1.3950 (19)	C18—H18C	0.9800
C1—C6	1.4006 (18)	C19—H19A	0.9800
C1—C7	1.4774 (18)	C19—H19B	0.9800
C2—C3	1.3954 (18)	C19—H19C	0.9800
C2—H2	0.9500	C20—H20A	0.9800
C3—C4	1.3982 (19)	C20—H20B	0.9800
C4—C5	1.3953 (19)	C20—H20C	0.9800
C5—C6	1.3950 (18)	O1M—C1M	1.4282 (18)
C6—H6	0.9500	O1M—H1M	0.8400
C7—C8	1.4057 (19)	C1M—H1M1	0.9800
C8—C9	1.4743 (18)	C1M—H1M2	0.9800
C9—C10	1.3936 (19)	C1M—H1M3	0.9800
C9—C14	1.3970 (18)		
			
N2—N1—C7	104.56 (11)	O6—C13—C12	115.88 (11)
N1—N2—N3	114.49 (11)	C14—C13—C12	120.27 (12)
N1—N2—H2N	124.0 (10)	C13—C14—C9	119.97 (12)
N3—N2—H2N	121.4 (11)	C13—C14—H14	120.0
N2—N3—C8	105.06 (11)	C9—C14—H14	120.0
C3—O1—C15	116.70 (10)	O1—C15—H15A	109.5
C4—O2—C16	113.71 (10)	O1—C15—H15B	109.5
C5—O3—C17	117.30 (11)	H15A—C15—H15B	109.5
C11—O4—C18	116.84 (10)	O1—C15—H15C	109.5
C12—O5—C19	116.01 (11)	H15A—C15—H15C	109.5
C13—O6—C20	116.87 (10)	H15B—C15—H15C	109.5
C2—C1—C6	120.65 (12)	O2—C16—H16A	109.5
C2—C1—C7	119.59 (12)	O2—C16—H16B	109.5
C6—C1—C7	119.77 (12)	H16A—C16—H16B	109.5
C1—C2—C3	119.53 (12)	O2—C16—H16C	109.5
C1—C2—H2	120.2	H16A—C16—H16C	109.5
C3—C2—H2	120.2	H16B—C16—H16C	109.5
O1—C3—C2	124.68 (12)	O3—C17—H17A	109.5
O1—C3—C4	115.06 (11)	O3—C17—H17B	109.5
C2—C3—C4	120.25 (12)	H17A—C17—H17B	109.5
O2—C4—C5	120.10 (11)	O3—C17—H17C	109.5
O2—C4—C3	120.05 (12)	H17A—C17—H17C	109.5
C5—C4—C3	119.81 (12)	H17B—C17—H17C	109.5
O3—C5—C6	124.27 (12)	O4—C18—H18A	109.5
O3—C5—C4	115.33 (11)	O4—C18—H18B	109.5
C6—C5—C4	120.39 (12)	H18A—C18—H18B	109.5
C5—C6—C1	119.32 (12)	O4—C18—H18C	109.5
C5—C6—H6	120.3	H18A—C18—H18C	109.5
C1—C6—H6	120.3	H18B—C18—H18C	109.5
N1—C7—C8	108.49 (11)	O5—C19—H19A	109.5
N1—C7—C1	121.12 (12)	O5—C19—H19B	109.5
C8—C7—C1	130.39 (12)	H19A—C19—H19B	109.5
N3—C8—C7	107.40 (11)	O5—C19—H19C	109.5
N3—C8—C9	121.97 (12)	H19A—C19—H19C	109.5
C7—C8—C9	130.48 (12)	H19B—C19—H19C	109.5
C10—C9—C14	120.46 (12)	O6—C20—H20A	109.5
C10—C9—C8	121.60 (12)	O6—C20—H20B	109.5
C14—C9—C8	117.94 (12)	H20A—C20—H20B	109.5
C9—C10—C11	119.35 (12)	O6—C20—H20C	109.5
C9—C10—H10	120.3	H20A—C20—H20C	109.5
C11—C10—H10	120.3	H20B—C20—H20C	109.5
O4—C11—C10	124.35 (12)	C1M—O1M—H1M	109.5
O4—C11—C12	115.03 (11)	O1M—C1M—H1M1	109.5
C10—C11—C12	120.62 (12)	O1M—C1M—H1M2	109.5
O5—C12—C13	121.12 (12)	H1M1—C1M—H1M2	109.5
O5—C12—C11	119.30 (12)	O1M—C1M—H1M3	109.5
C13—C12—C11	119.29 (12)	H1M1—C1M—H1M3	109.5
O6—C13—C14	123.85 (12)	H1M2—C1M—H1M3	109.5
			
C7—N1—N2—N3	−0.41 (15)	N2—N3—C8—C9	−175.64 (12)
N1—N2—N3—C8	0.07 (16)	N1—C7—C8—N3	−0.54 (15)
C6—C1—C2—C3	−0.27 (19)	C1—C7—C8—N3	−179.60 (13)
C7—C1—C2—C3	−179.82 (12)	N1—C7—C8—C9	174.91 (13)
C15—O1—C3—C2	3.37 (19)	C1—C7—C8—C9	−4.1 (2)
C15—O1—C3—C4	−177.50 (12)	N3—C8—C9—C10	−47.04 (19)
C1—C2—C3—O1	178.74 (12)	C7—C8—C9—C10	138.07 (15)
C1—C2—C3—C4	−0.36 (19)	N3—C8—C9—C14	132.31 (14)
C16—O2—C4—C5	85.75 (15)	C7—C8—C9—C14	−42.6 (2)
C16—O2—C4—C3	−96.54 (15)	C14—C9—C10—C11	−0.87 (19)
O1—C3—C4—O2	4.99 (18)	C8—C9—C10—C11	178.47 (12)
C2—C3—C4—O2	−175.83 (11)	C18—O4—C11—C10	−4.16 (18)
O1—C3—C4—C5	−177.30 (11)	C18—O4—C11—C12	175.12 (12)
C2—C3—C4—C5	1.9 (2)	C9—C10—C11—O4	−179.85 (12)
C17—O3—C5—C6	8.57 (19)	C9—C10—C11—C12	0.91 (19)
C17—O3—C5—C4	−172.45 (12)	C19—O5—C12—C13	−72.53 (17)
O2—C4—C5—O3	−4.09 (18)	C19—O5—C12—C11	113.70 (14)
C3—C4—C5—O3	178.19 (12)	O4—C11—C12—O5	−4.85 (18)
O2—C4—C5—C6	174.92 (12)	C10—C11—C12—O5	174.46 (11)
C3—C4—C5—C6	−2.79 (19)	O4—C11—C12—C13	−178.74 (11)
O3—C5—C6—C1	−178.92 (12)	C10—C11—C12—C13	0.6 (2)
C4—C5—C6—C1	2.16 (19)	C20—O6—C13—C14	6.21 (18)
C2—C1—C6—C5	−0.6 (2)	C20—O6—C13—C12	−173.47 (11)
C7—C1—C6—C5	178.93 (12)	O5—C12—C13—O6	3.80 (18)
N2—N1—C7—C8	0.56 (15)	C11—C12—C13—O6	177.57 (11)
N2—N1—C7—C1	179.72 (12)	O5—C12—C13—C14	−175.89 (12)
C2—C1—C7—N1	−34.27 (18)	C11—C12—C13—C14	−2.11 (19)
C6—C1—C7—N1	146.18 (13)	O6—C13—C14—C9	−177.50 (12)
C2—C1—C7—C8	144.69 (15)	C12—C13—C14—C9	2.16 (19)
C6—C1—C7—C8	−34.9 (2)	C10—C9—C14—C13	−0.66 (19)
N2—N3—C8—C7	0.28 (15)	C8—C9—C14—C13	179.98 (12)

### Hydrogen-bond geometry (Å, º)

**Table:**

*D*—H···*A*	*D*—H	H···*A*	*D*···*A*	*D*—H···*A*
N2—H2*N*···O1*M*	0.936 (17)	1.797 (18)	2.7303 (15)	174.2 (16)
O1*M*—H1*M*···N3^i^	0.84	1.97	2.8101 (15)	177

Symmetry code: (i) −*x*+2, −*y*+1, −*z*+1.
