# Supplementary figures and images for: Crystal structure of 4,5-bis­(3,4,5-tri­meth­oxy­phen­yl)-2H-1,2,3-triazole methanol monosolvate
Source: Acta Crystallogr Sect E Struct Rep Online. 2014 Sep 24;70(Pt 10):o1128–9. doi: 10.1107/S1600536814020911 (PMC4257191; doi:10.1107/S1600536814020911)

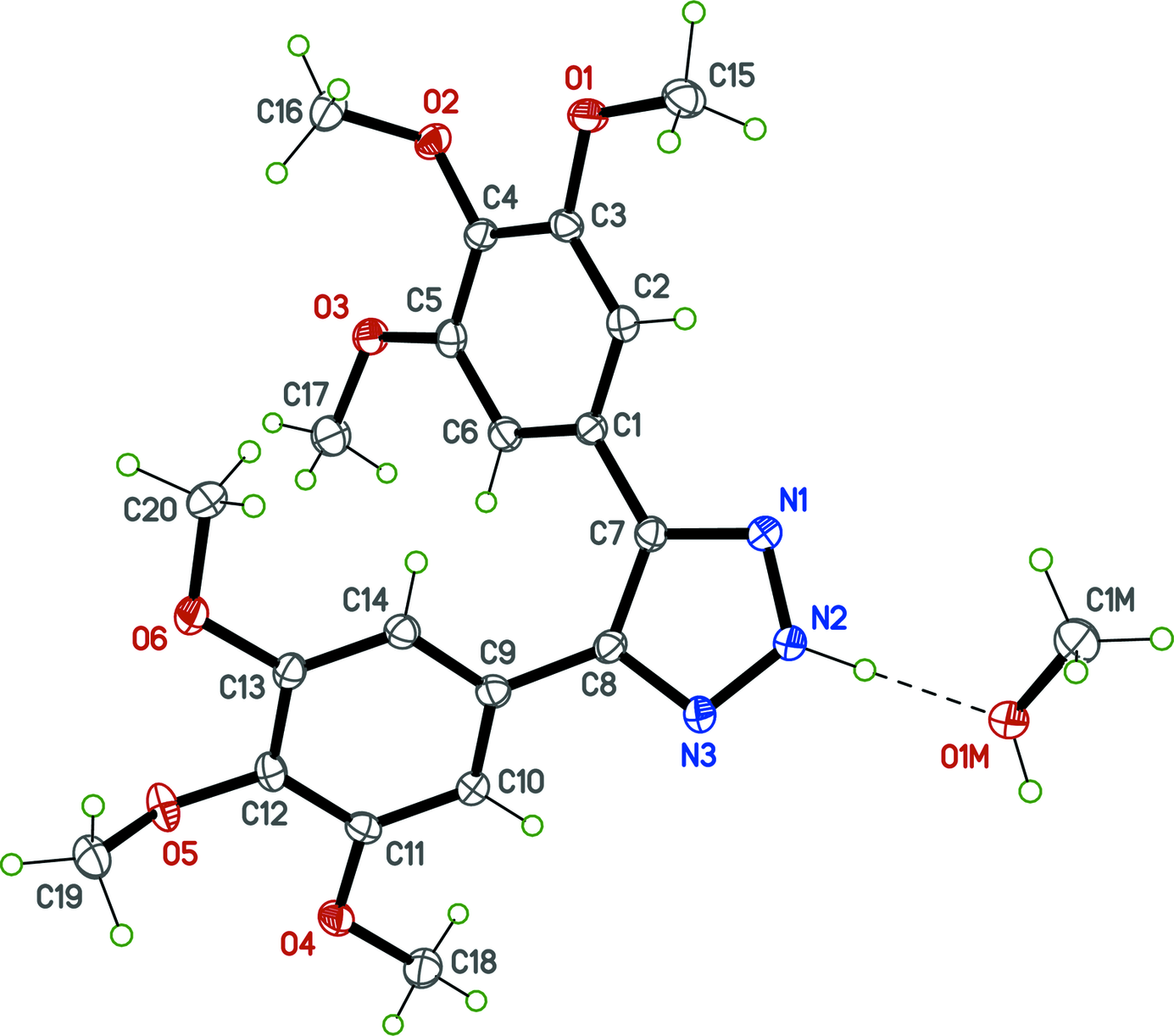

Supplement: Supplementary file 4 [file e-70-o1128-fig1.tif]
